# The Influence of Nutritional Status on Brain Development: Benefits of Exclusive Breastfeeding

**DOI:** 10.3390/pediatric16030061

**Published:** 2024-08-24

**Authors:** Ellen Schavarski Chade, Odonis Rocha Júnior, Nathalia Marçallo Peixoto Souza, Aline Jacoski de Oliveira Krüger da Silva, Luana Mota Ferreira, Jéssica Brandão Reolon, Juliana Sartori Bonini, Fabiane Gomes de Moraes Rego, Marcel Henrique Marcondes Sari

**Affiliations:** 1 Departamento de Farmácia, Universidade Estadual do Centro-Oeste (UNICENTRO), Guarapuava 85040-167, Paraná, Brazil; ellenchade2015@gmail.com (E.S.C.); odonisjrrocha@gmail.com (O.R.J.); jessica_breolon@yahoo.com.br (J.B.R.); juliana.bonini@gmail.com (J.S.B.); 2 Programa de Pós-Graduação em Ciências Farmacêuticas, Universidade Federal do Paraná (UFPR), Curitiba 80210-170, Paraná, Brazil; nathaliamarcallo@gmail.com (N.M.P.S.); fgmrego@gmail.com (F.G.d.M.R.); 3 Departamento de Nutrição, Universidade Estadual de Ponta Grossa (UEPG), Ponta Grossa 84030-900, Paraná, Brazil; aline.nutri.ak@gmail.com; 4 Departamento de Análises Clínicas, Universidade Federal do Paraná (UFPR), Curitiba 80210-170, Paraná, Brazil

**Keywords:** breast milk, neurodevelopment, nutrient, cognitive development

## Abstract

Background: This study aimed to conduct a narrative review approaching the effects of exclusive breastfeeding on neuropsychomotor development. The goal was to provide evidence-based knowledge to inform healthcare practices and policies and promote optimized infant feeding strategies. Methods: Our study reviewed the relevant literature from May and June 2024, covering the publication period between 2013 and 2024. The PubMed database was utilized and searched for articles using keywords such as “Brain”, “Growth”, “Development”, and “Breastfeeding”, employing Boolean operators such as “AND”, “OR”, and “NOT.” Results: Our search initially screened 15,412 studies, resulting in 600 articles. Eleven studies met our inclusion criteria and provided relevant information on the topic. Several studies have shown that exclusive breastfeeding and its duration are beneficial for neural development. Research suggests that breastfeeding improves brain architecture, white matter development, and cognitive performance. Additionally, studies indicate that the mother’s intake of omega-3 fatty acids can enhance infant brain development, and specific micronutrients in breast milk, such as myo-inositol, may contribute to neural connectivity. Some findings also suggest that the child’s sex may play a role in how breast milk benefits the brain. Furthermore, there is evidence of the strong influence of epigenetic compounds on the neurodevelopmental benefits of exclusive breastfeeding. Conclusions: This narrative review revealed findings that indicate breast milk has a positive impact on brain development. This emphasizes that breast milk has a positive impact on brain development. It underscores the importance of conducting additional research to understand how breastfeeding specifically influences neurodevelopment.

## 1. Introduction

Numerous studies since the mid-1980s have highlighted the importance of breastfeeding and its numerous benefits for babies. Breastfeeding is considered one of the main determinants of infant health in the short and long term. It can prevent acute infections and reduce the risk of chronic diseases, as well as provide nutritional, psychological, immunological, cognitive, neural, and emotional benefits [1,2,3,4].

Breast milk is a complete food that provides the necessary nutrition for the child’s needs. The recommended duration for exclusive breastfeeding is six months, followed by complementary breastfeeding for at least two years [1]. During the first six months, there is no need to introduce other foods such as tea, juice, or water [5].

Brain development begins during pregnancy and reaches maturity around the age of three. Proper nutrition is crucial for healthy brain development early in life and can have long-lasting effects on cognitive and mental health [4]. Research suggests that breastfeeding has specific benefits for infant brain development, such as increased myelination, differences between gray and white matter, and increased hippocampal volume in exclusively breastfed infants and young children. These characteristics reflect better cognitive and neural development [6].

While it is widely known that exclusive breastfeeding provides numerous benefits for the protection and development of the child, infant formula remains a popular substitute. This is concerning because the formula does not provide the same nutrients as breast milk and can impair the child’s maximum developmental potential [7]. Globally, the rate of exclusive breastfeeding in children under six months is only 41%, while the World Health Organization (WHO) aims to reach the target of 70% by 2030. These statistics underline the need for new strategies to promote exclusive breastfeeding [8,9]. One such approach is Evidence-Based Policy, which uses scientific knowledge to increase the effectiveness of public health policies [10,11].

This narrative review synthesizes data from various research instruments to explore the impact of diet and nutritional status on child neurodevelopment. It focuses on the crucial role of breastfeeding in children’s cognitive and behavioral development and seeks evidence of its benefits. The results of this study may lead to the development of interventions promoting exclusive breastfeeding, contributing to our understanding of how early nutritional choices can shape long-term development, and suggesting avenues for further research. The research examines studies that highlight the relationship between breastfeeding, cognition, executive function, and behavior.

## 2. Materials and Methods

This article aims to synthesize knowledge and incorporate the applicability of results obtained in studies on a specific topic or issue, through a narrative review of the literature, in which relevant studies were systematically located and subsequently discussed, presenting their underlying conclusions.

### 2.1. Search Strategy

The search was conducted between May and June 2024, using the PubMed database. We used the keywords “Brain” AND “Growth” OR “Development” AND “Breastfeeding”, which were combined using the Boolean operators “AND” and “NOT”.

### 2.2. Eligibility Criteria and Study Selection Criteria

The inclusion criteria for this study encompassed systematic reviews, narrative reviews, and observational studies that focused on the impact of exclusive breastfeeding on neurocognitive development in infants and young children. Eligible studies were required to be published in English. The research needed to provide evidence of the benefits of exclusive breastfeeding for brain development and address specific mechanisms through which breastfeeding influences neurocognitive outcomes, such as the role of micronutrients, bioactive compounds, or epigenetic factors. Studies were excluded if they specifically addressed diseases unrelated to neurodevelopment, focused on maternal health rather than child development, or discussed interventions to promote breastfeeding instead of its effects on neurocognitive development.

### 2.3. Data Extraction

The relevant data present in the selected articles were grouped in a table, and the following information was extracted from each article: basic characteristics such as author and year of publication, study population (quantity, age range, and full-term or preterm birth), evaluation methodology (imaging, electroencephalography, questionnaires, and scales), comparative evaluation between children fed exclusively with breast milk and children fed with formulas infants, analysis of the relationship between breastfeeding duration and neurocognitive benefits, and association of nutrients present in breast milk with better child neurodevelopment.

The authors performed a detailed analysis and discussed the primary results of the studies, defining any relevant concepts and conclusions that can be drawn from them.

## 3. Results

**Figure 1:**
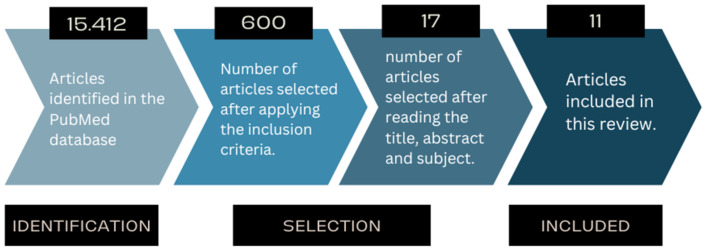
Flowchart of the steps taken to select the articles.

After conducting our initial search, we screened a total of 15,412 studies and identified 600 relevant articles. Out of these, 583 articles were excluded because they did not meet the inclusion criteria, leaving seventeen relevant studies. After a careful and detailed reading, six articles were removed, leaving eleven that provided valuable and pertinent information on the topic at hand. These findings are depicted in Figure 1.

This research delved into various publications that utilized a qualitative and integrative approach to explore the intricate relationship between nutrition and brain development published between 2013 and 2024. Among these publications, three specifically delved into the critical review of clinical studies examining the correlation between diet and neurodevelopment (27%—3/11) [12,13,14]. Furthermore, five papers relied on magnetic resonance imaging (MRI) techniques (45%—5/11) [15,16,17,18,19], while one article monitored brain electrical activity through electroencephalogram (EEG) to gather valuable data on food intake and its impact on brain development (9%—1/11) [20]. Another study utilized questionnaires to assess elements such as cognitive function and non-verbal intelligence in relation to breastfeeding patterns (9%—1/11) [21]. Additionally, one study took a unique approach by culturing neurons in vitro and subjecting them to breast milk constituents to evaluate synapses (9%—1/11) [22].

The observational studies included samples of preterm infants, full-term infants aged between 1 and 82 weeks, and children aged between 2 and 9 years. Additionally, 64% (7/11) of the authors of the analyzed articles were affiliated with American institutions [12,14,15,16,19,20,22], 9% (1/11) were from Canadian research institutions [18], another 9% (1/11) were from Chilean institutions [17], 9% (1/11) were from Japanese institutions [21], and, finally, 9% (1/11) were from Greek institutions [13]. All the abovementioned data are summarized in Table 1.

The review article examines the impact of exclusive breastfeeding on neuropsychomotor development. It emphasizes the role of breastfeeding in enhancing brain development and cognitive performance. The study highlights the influence of specific micronutrients in breast milk, epigenetic compounds, and the child’s sex in shaping these benefits. Lastly, to the best of our knowledge, our revision approaches a seldom-discussed topic. Its findings provide valuable insights for healthcare practices and policies, necessitating further research into the long-term neurodevelopmental benefits of exclusive breastfeeding.

The research highlights a positive link between breastfeeding and enhanced brain development, although the exact mechanisms behind this connection are not fully understood. Recent studies have revealed that breast milk can support neural growth, connections, and myelination. It is believed that the nutritional composition of breast milk plays a significant role in providing these benefits, with nutrients like myoinositol sugar, human milk oligosaccharides (HMOs), and long-chain polyunsaturated fatty acids such as docosahexaenoic and arachidonic acid contributing to brain development. Additionally, the high cholesterol content in breast milk is thought to aid cognitive development by providing a readily available supply for myelination. These findings are supported by studies conducted by Berger (2023) [12], Morton (2020) [15], Niu et al. (2020) [17], and Paquette (2023) [22]. 

### 3.1. Potential Mechanisms Involved in the Positive Association between Breastfeeding and Neurodevelopment

It is known that the development and maintenance of brain connectivity are influenced by genetics, environment, and experience. Although the role of bioactive dietary compounds in this process is not fully understood, diet is an important environmental factor. Consequently, various studies aim to assess the impact of micronutrients in breast milk on neurodevelopment. Human milk oligosaccharides (HMOs) are the third most abundant solid component of milk. They are complex and structurally diverse carbohydrates with various physiological functions that can affect brain maturation and neurodevelopment. Several studies have shown a significant connection between exposure to HMOs during breastfeeding and neurodevelopmental outcomes in infants, especially when exposed to higher concentrations of specific and total fucosylated and sialylated HMOs during the recommended exclusive breastfeeding period. These studies provide important observational evidence of the influence of dietary factors on neurological development [12].

In a study conducted by Paquette (2023) [22], the influence of a bioactive dietary compound found in human milk on brain development was evaluated. The study focused on the carbocyclic carbohydrate myo-inositol and its role in promoting brain connectivity using rodent and mouse neuron models. The study found that myo-inositol significantly promoted the development of synaptic specializations in the neurons of the hippocampus in rats. Additionally, the study demonstrated that myo-inositol increased the synaptogenic effect of Neuroligin 1 and promoted the induction of presynaptic sites.

The study also evaluated the abundance of excitatory synaptic sites in mature brain tissue and found that exposure to myo-inositol increased the number of synapses. Furthermore, the study determined that myo-inositol concentration peaks at the beginning of lactation, which coincides with the period when neuronal connections form rapidly in a baby’s brain. The study emphasized the importance of breast milk micronutrients for infant neurodevelopment, especially during the first years of life [22].

The relationship between breast milk consumption and enhanced brain development may be influenced by gene modulations provided by bioactive components present in human milk. A study by Gialeli et al. (2023) [13] aimed to analyze the association between breastfeeding and neurodevelopment through epigenetics. The study highlighted three bioactive components present in breast milk that could be involved in this association: micro-RNAs (miRNAs), stem cells, and long non-coding RNAs (lncRNAs). These components are abundant in breast milk and can cross the blood–brain barrier, potentially impacting brain development. For example, different miRNAs in breast milk regulate neural cell proliferation and differentiation, and synaptic plasticity. Long non-coding RNAs play a crucial role in neurogenesis and synaptogenesis, while stem cells have demonstrated the ability to reach brain tissue and differentiate into neuronal and glial cells. The presence of these compounds in high concentrations in breast milk suggests an alternative mechanism by which breastfeeding supports infant neurodevelopment [13].

In a recent study conducted by Morton (2020) [15], researchers aimed to explore how maternal nutrient intake affects the brain development of exclusively breastfed full-term babies. The study involved 92 infants, and magnetic resonance imaging techniques were used to assess the babies’ brain development. Maternal nutrient intake was evaluated through standardized food frequency questionnaires. The results showed that higher intake of omega-3 fatty acids by the mothers was positively associated with increased volumes in specific areas of the babies’ brains, such as the sub-regions of the frontal cortex and corpus callosum. Notably, these benefits were not observed in infants who were fed with infant formula. The increased brain volume is believed to be crucial for greater brain mass, which is important for activities planning and execution, and communication between the two hemispheres of the brain. These findings contribute to our understanding of the potential benefits of exclusive breastfeeding on child neurodevelopment. They may also provide valuable insights for developing interventions to optimize child neurodevelopment [15].

### 3.2. Neurocognitive Benefits Observed in Exclusively Breastfed Children

The study by Deoni et al. (2013) [16] supports previous findings in brain imaging studies showing a link between increased white matter, subcortical gray matter, and cortical thickness in the parietal lobe with a higher IQ in children who were breastfed compared to formula-fed babies. The study involved 133 children categorized into three groups: (a) exclusively breastfed (85 children), (b) exclusively formula-fed (38 children), and (c) breastfed and formula-fed (51 children). The children were assessed using magnetic resonance imaging with the mcDESPOT white matter imaging technique and the Mullen Early Learning Scales. The results indicate that breastfeeding is associated with better brain growth and development and that prolonged breastfeeding, in particular, is associated with better white matter structure in two brain regions that are anatomically related to better cognitive and behavioral performance. The study found that children who were exclusively breastfed showed more significant improvements in white matter development and cognitive performance compared to those who were exclusively formula-fed. The group of children who received a combination of formula and breast milk showed milder positive results, with their developmental progress falling between the exclusively breastfed and exclusively formula-fed groups. This demonstrates that exclusive breastfeeding is specifically linked to better outcomes in brain development and cognitive performance. While the exact reasons for these benefits are not fully understood, the study suggests that long-chain polyunsaturated fatty acids or other essential fatty acids in breast milk may play a role in these advantages [16].

According to a study aiming to identify the most effective strategies for enhancing early brain development and cognitive functioning in children, providing adequate nutrition during the first 1000 days (about 2 and a half years) of life is crucial. Postnatal growth appears to play a significant role in subsequent neurodevelopment, particularly in prematurely born babies [14]. Another study involved the evaluation of 50 premature babies, 30 of whom were exclusively breastfed, while 20 were fed with infant formula. The study aimed to investigate the possible association between feeding and brain development. The infants underwent resting-state functional magnetic resonance imaging (fMRI), which examined the 3D spatiotemporal architecture of the temporal brain network. The results indicated that breast-feed preterm infants demonstrated higher overall temporal efficiency than those fed with formula. Specifically, localized elevations of temporal nodal properties were observed in the right temporal gyrus and bilateral caudate. These findings correlate with the measurement of localized temporal information transmission, showing advantages in brain growth at both the global and regional levels [17].

Several studies have shown that breastfed babies tend to perform better on cognitive tests compared to formula-fed babies. This difference is associated with higher concentrations of white and gray matter in the brain, which can be measured using MRI. To further investigate the impact of feeding on cognitive development, one study compared the brain activity of exclusively breastfed babies with formula-fed babies using electroencephalography (EEG). EEG measures neuronal activity and can provide information about cognitive development. The study assessed 536 babies between the ages of 2 and 6 months and analyzed the EEG readings to identify differences in the frequency bands associated with cognitive processes. The results showed significant changes in neural activity in babies at both 2 and 6 months, suggesting that feeding may have a specific effect on neurodevelopment during these critical periods. In addition, improvements were observed in the gamma and beta frequency bands, indicating the development of the GABAergic system and its role in the functional architecture of the neuronal network. In contrast, exclusively formula-fed infants had lower white matter development, specifically in regions associated with language, motor skills, and cognitive functions, and scores on cognitive performance tests were lower for this group. It is worth noting that the observed differences in neural development also correlated with better motor and cognitive assessment scores in exclusively breastfed infants [20].

The evidence suggests that exclusively breastfeeding during the first months after birth plays a crucial role in brain development and can have lifelong impacts. Researchers conducted a study to examine the effects of exclusive breastfeeding and its duration on neurodevelopment by evaluating 86 children aged 2 to 6 years. They used magnetic resonance imaging, specifically the diffusion tensor technique (DTI), to assess brain microstructure, including myelination, axonal coating, and fiber interpretation. The results indicated that longer periods of breastfeeding are linked to greater fractional anisotropy, suggesting better white matter integrity. Interestingly, these effects were more prominent in boys compared to girls, indicating a possible gender-specific influence on the benefits [18]. This finding corroborates a previous study from 2014 which aimed to determine whether breastfeeding has a specific impact on brain development depending on gender. This study evaluated 56 healthy children aged 7 to 8, according to gender, using magnetic resonance imaging to assess white matter development; additionally, brain structure, cognitive development, and myelination were assessed according to the feeding pattern of the children included in the study, in order to establish a comparison between exclusively breastfed children and children fed infant formula. The results showed that boys who were exclusively breastfed during infancy had better white matter development, but this association was not observed in girls. In relation to the feeding pattern, exclusively breastfed children showed advantages in relation to cognitive development, myelination, and behavioral outcomes when compared to formula-fed children [19]. While there is evidence supporting the impact of nutrition on brain development and cognitive function, the specific neurophysiological mechanisms through which breastfeeding affects this development are still not fully understood. Therefore, new findings that shed light on these mechanisms should be considered for informing future studies.

In 2023, Lovcevic and collaborators [21] conducted a study to determine the impact of exclusive breastfeeding on the cognitive development of children from early childhood to mid-adolescence. Using data from the “Growing Up in Australia” longitudinal cohort study, the researchers assessed cognitive outcomes, language skills, and non-verbal intelligence at different ages. The study found that longer breastfeeding duration was associated with better language skills in children aged 5 to 9 years and higher non-verbal intelligence in children aged 7 to 11 years. However, no significant relationship was found between breastfeeding duration and executive functions at 15 years. The study concluded that prolonged breastfeeding contributes to improved non-verbal language and cognitive skills in early and middle childhood but does not impact executive function in mid-adolescence [21].

## 4. Discussion

The findings from the reviewed studies support the existing data from the literature, forming a solid body of evidence indicating that breast milk is crucial for promoting healthy brain development. While the exact reasons behind these benefits are still under investigation, it is reasonable to assume that the positive impact of breastfeeding on neurodevelopment is not solely attributable to a single factor, but rather to the various components present in breast milk.

The impact of exclusive breastfeeding on neurocognitive development is a multifaceted topic, as evidenced by the diverse methodologies and outcomes of the studies reviewed. Berger et al. (2023) underscored the significant association between human milk oligosaccharides and improved cognitive, language, and motor skills in full-term infants [12]. This narrative review highlights how fucosylated and sialylated oligosaccharides in breast milk contribute positively to neurodevelopmental outcomes, suggesting that the complex carbohydrates in breast milk play a crucial role in cognitive growth. Similarly, Paquette et al. (2023) identified myo-inositol as a key bioactive component in breast milk that supports neuronal connectivity [22]. Their research demonstrated that myo-inositol enhances synaptic development, emphasizing the importance of specific nutrients in fostering brain development during critical early life periods.

In addition to specific nutrients, broader studies have explored the benefits of exclusive breastfeeding on brain structure and function. Morton et al. (2019) utilized magnetic resonance imaging to reveal that higher maternal intake of omega-3 fatty acids was associated with increased brain volumes in infants, particularly in regions critical for cognitive functions [15]. This finding aligns with previous research indicating that essential fatty acids in breast milk contribute to better brain development. On the other hand, Deoni et al. (2013) [16] and Kar et al. (2021) [18] provided evidence that exclusive breastfeeding is linked to enhanced white matter development and cognitive performance. Their studies demonstrated that breastfed children exhibit superior white matter structure and cognitive abilities compared to formula feeding, reinforcing that breastfeeding supports brain maturation and cognitive growth.

The role of breastfeeding in shaping brain network architecture and cognitive development has also been highlighted in recent studies. Niu et al. (2020) observed improved brain network organization in prematurely born infants exclusively breastfed, suggesting that breastfeeding positively affects brain network efficiency [17]. Furthermore, Gilbreath et al. (2023) found that breastfed infants showed higher beta and gamma activity in the frontal cortex, associated with advanced cognitive functions [20]. These findings support the hypothesis that breastfeeding influences brain electrical activity and cognitive performance. Additionally, Lovcevic (2023) found that longer breastfeeding duration was associated with enhanced language skills and nonverbal intelligence, though it did not affect executive functions [21]. This study adds to the evidence that extended breastfeeding contributes to specific aspects of cognitive development, underscoring the need for continued research into how breastfeeding duration impacts various cognitive domains.

The findings from the reviewed studies strongly support the existing literature, indicating that exclusive breastfeeding plays a vital role in promoting healthy brain development. Our review focused on studies demonstrating positive associations between breastfeeding and neurocognitive outcomes. However, it is essential to consider both supporting and contrasting evidence and place these findings within a broader context.

Several studies that were not included in our initial review have examined the effects of exclusive breastfeeding on neurodevelopment, yielding varying results. For example, a meta-analysis by Horta et al. (2015) [23] discovered that while breastfeeding was linked to enhanced cognitive performance in childhood, the impact was modest and the benefits may decrease over time. This implies that while breastfeeding has a positive influence on early brain development, other factors such as genetic predispositions, environmental influences, and ongoing nutritional support after breastfeeding also significantly contribute to long-term cognitive results.

Furthermore, the literature also includes studies with neutral or negative findings regarding the impact of breastfeeding on neurodevelopment. For example, a study by Brion et al. (2011) [24] suggested that after adjusting for socioeconomic status and parental IQ, the cognitive advantages attributed to breastfeeding were less pronounced, indicating that confounding factors might partly explain the observed benefits. Similarly, Der et al. (2006) [25] found that the association between breastfeeding and cognitive development was attenuated when controlling for maternal intelligence, highlighting the complexity of isolating breastfeeding as a sole contributor to neurodevelopment.

In addition to cognitive outcomes, the influence of breastfeeding on emotional and behavioral development has also been explored. A study by Kramer et al. (2008) [26], part of the PROBIT trial, reported no significant differences in behavioral outcomes between breastfed and formula-fed children, challenging the notion that breastfeeding universally enhances all aspects of neurodevelopment. This further emphasizes the need for caution when interpreting the benefits of breastfeeding, as its impact may vary across different domains of neuropsychomotor development.

Our review identified specific nutrients in breast milk, such as omega-3 fatty acids and myo-inositol, associated with enhanced neural connectivity and cognitive performance. However, it is crucial to recognize that the biological mechanisms underpinning these associations are still not fully understood. Other research has pointed to the role of epigenetic modifications, as noted by Melchior et al. (2017) [27], who found that breastfeeding might influence gene expression related to brain development. This highlights the complex interplay between nutrition, genetics, and environmental factors in shaping neurodevelopmental outcomes.

Moreover, while our review highlighted the positive impacts of exclusive breastfeeding, it is essential to consider the potential for publication bias in the literature. Studies with positive findings are more likely to be published, which could skew the overall perception of breastfeeding benefits. Future research should aim to include a more balanced representation of both positive and negative findings to provide a clearer picture of the actual effects of breastfeeding on neurodevelopment.

The results of the current review are positive, but the studies we analyzed have some limitations. For instance, when the studies included infants who were fed infant formula, they did not specify the composition and volume of the formula. Another potential factor that was not reported is the exposure of babies to antibiotic drugs, which can directly interfere with the intestinal microbiota, affecting nutrient absorption from breast milk. Future research on the advantages of breast milk for child development should include longitudinal studies with large and diverse samples, considering factors such as socioeconomic status, parental education, and maternal health. It is also important to standardize the methodologies used for cognitive and behavioral assessments. Furthermore, there is a need for mechanistic studies and the identification of biochemical markers to understand the biological basis of this association. We acknowledge that the review methodology used in our study may have limitations, as it lacks standardized search criteria, which could affect reproducibility. Additionally, we did not use quantitative techniques to combine data from different studies, which may limit our ability to statistically assess the overall relationship of the effects. We also did not consistently assess the quality of the studies included.

Understanding the connection between exclusive breastfeeding and brain development is crucial for developing evidence-based educational strategies that promote exclusive breastfeeding. This has significant health, neurobiological, and public health implications for parents, caregivers, guardians, and professionals [10,12,14,16,17].

## 5. Conclusions

This review provides compelling evidence that exclusive breastfeeding is associated with enhanced brain development and cognitive function in infants compared to formula feeding. Our analysis of studies reveals that exclusively breastfed infants exhibit stronger neural development, improved brain white matter, and superior cognitive performance. These findings underscore the significant role of breastfeeding in supporting optimal neurodevelopment.

The evidence highlights several key mechanisms breast milk may contribute to these benefits, including bioactive compounds such as human milk oligosaccharides, myo-inositol, and long-chain polyunsaturated fatty acids. However, the precise biological pathways and mechanisms remain to be fully elucidated. While our review demonstrates the positive impact of breastfeeding on neurodevelopment, it also identifies a critical need for further research to explore and understand these underlying mechanisms more comprehensively.

However, while the evidence supports the benefits of exclusive breastfeeding for neuropsychomotor development, it is imperative to approach these findings with a nuanced perspective. The interplay between breastfeeding and genetic, environmental, and socioeconomic factors complicate the ability to attribute cognitive and developmental outcomes solely to breastfeeding. Therefore, future studies should adopt more rigorous methodologies, control for potential confounders, and include a broader range of outcomes better to understand the comprehensive impact of breastfeeding on child development.

Therefore, the findings reinforce the importance of promoting exclusive breastfeeding as a vital aspect of infant care and nutrition. Future research should focus on elucidating the specific components of breast milk that contribute to neurodevelopmental advantages and exploring the potential for tailored nutritional interventions to optimize brain development. This will help to inform healthcare practices and policies better aimed at maximizing infants’ developmental outcomes.

## Figures and Tables

**Table 1:** Articles identified in the PubMed database, according to authors and year of publication, objectives, design and methods, and results.

Author and Year of Publication	Objective	Sample	Methodology	Results
**Berger et al., (2023) [12]**	Identify and synthesize studies on associations of oligosaccharide exposure during childhood with neurodevelopmental outcomes.	29 articles.	Narrative review.	In full-term infants, fucosylated and sialylated oligosaccharides were positively associated with cognitive, language, and motor skills.
**Gialeli et al., (2023) [13]**	Discuss the potential epigenetic role of miRNAs, RNAs, stem cells, and the human milk microbiome in the neurodevelopment of infants.	122 articles.	Narrative review.	The potential epigenetic effects of breast milk through its bioactive components such as non-coding RNA and stem cells could explain the association between breast milk and improved neurodevelopmental outcomes.
**Black, (2018) [14]**	To assess the impact of nutrition on brain and cognitive development.	Not specified.	Narrative review.	Improved myelination and increased neural plasticity in exclusively breastfed infants.
**Morton et al., (2019) [15]**	To relate maternal nutrients to infant brain morphometry at the regional and voxel levels.	92 babies aged 1 to 7 weeks.	Brain evaluation by magnetic resonance imaging and application of a feeding pattern questionnaire.	Positive relationship between maternal intake of omega-3 fatty acids and increased voxel volumes of the infant brain, predominantly in the frontal lobes and corpus callosum.
**Deoni et al., (2013) [16]**	To investigate the influence of breastfeeding on the early development of white matter and myelin.	133 healthy children from 10 months to 4 years of age.	Magnetic resonance imaging brain assessment and application of the Mullen Early Learning Scale.	Improved brain white matter development and improved cognitive performance in exclusively breastfed children.
**Niu et al., (2020) [17]**	To explore the influence of breastfeeding on the reorganization of brain networks.	50 premature infants at ~33 weeks.	Brain evaluation by magnetic resonance imaging and evaluation of feeding pattern by questionnaires.	Exclusively breastfed infants showed improved brain network architecture at the global and regional levels.
**Kar et al., (2021) [18]**	To investigate whether exclusive breastfeeding and its duration is associated with the microstructure of the brain’s white matter.	85 typically developing children between 2 and 6 years of age.	Magnetic resonance imaging (MRI) brain assessment and application of questionnaires to investigate feeding patterns.	Greater development of the global and regional white matter microstructure in exclusively breastfed infants when compared to infant formula recipients.
**Ou et al., (2014) [19]**	To establish a comparative effect between brain white matter development between exclusively breastfed infants and infant formula-fed infants.	56 healthy children aged 7 to 8 years.	Magnetic resonance imaging and application of the following scales: Reynolds Intellectual Assessment Scale (RIAS) and Clinical Assessment of Language Fundamentals (CELF-4).	Significantly higher volume of white matter in exclusively breastfed men when compared to those who received infant formula.
**Gilbreath et al., (2023) [20]**	To determine whether differences in brain activity arose between children with different diet patterns.	536 infants between the ages of 2 and 6 months.	Analysis of brain electrical activity by electroencephalogram (EEG).	Beta and gamma were significantly higher in breastfed infants, with regional differences in the frontal cortex. Also, higher beta in the frontal and temporal lobes.
**Lovcevi, (2023) [21]**	To evaluate the dose-response association between breastfeeding duration and cognitive abilities in children aged 5 to 15 years.	Variable according to the research question.	Application of the Peabody Picture Vocabulary Test (to assess language skills), the Matrix Reasoning Subtest of the Wechsler Intelligence Scale (to assess non-verbal intelligence) and the Cogstate Cognitive Testing Battery (to assess cognitive functions). Breastfeeding patterns were assessed using prospective questionnaires.	A longer breastfeeding duration was significantly associated with greater language skills and nonverbal intelligence. No significant relationship was evaluated between breastfeeding duration and cognitive performance.
**Paquette et al., (2023) [22]**	Understand the impact of human milk on the infant brain and identify myo-inositol as a component of breast milk that promotes the formation of neuronal connections.	Not specified.	Determination of myo-inositol concentration in milk samples by HPAEC-PAD. In vitro culture of neurons added to myo-inositol whose synaptic analyses were performed by immunostaining followed by confocal microscopy.	The study reveals substantial benefits of myo-inositol for postnatal brain connectivity, highlighting breast milk as a dynamic source of bioactive compounds that support neurodevelopment.
